# Migration of *Paraburkholderia terrae* BS001 Along Old Fungal Hyphae in Soil at Various pH Levels

**DOI:** 10.1007/s00248-017-1137-1

**Published:** 2018-01-10

**Authors:** Pu Yang, Renata Oliveira da Rocha Calixto, Jan Dirk van Elsas

**Affiliations:** Microbial Ecology, Groningen Institute for Evolutionary Life Sciences, University of Groningen, Nijenborgh 7, 9747 AG Groningen, The Netherlands

**Keywords:** Bacterial motility, Soil pH, Soil moisture, Mycosphere

## Abstract

The movement of bacterial cells along with fungal hyphae in soil (the mycosphere) has been reported in several previous studies. However, how local soil conditions affect bacterial migration direction in the mycosphere has not been extensively studied. Here, we investigated the influence of two soil parameters, pH and soil moisture content, on the migration, and survival, of *Paraburkholderia terrae* BS001 in the mycosphere of *Lyophyllum* sp*.* strain Karsten in microcosms containing a loamy sand soil. The data showed that bacterial movement along the hyphal networks took place in both the “forward” and the “backward” directions. Low soil pH strongly restricted bacterial survival, as well as dispersal in both directions, in the mycosphere. The backward movement was weakly correlated with the amount of fungal tissue formed in the old mycelial network. The initial soil moisture content, set at 12 versus 17% (corresponding to 42 and 60% of the soil water holding capacity), also significantly affected the bacterial dispersal along the fungal hyphae. Overall, the presence of fungal hyphae was found to increase the soil pH (under conditions of acidity), which possibly exerted protective effects on the bacterial cells. Finally, we provide a refined model that describes the bacterial migration patterns with fungal hyphae based on the new findings in this study.

**Electronic supplementary material:**

The online version of this article (10.1007/s00248-017-1137-1) contains supplementary material, which is available to authorized users.

## Introduction

Bacterial migration along with mycelium-forming organisms in soil has been extensively studied in recent years. *Pseudomonas putida* PpG7 was reported to be able to disperse along with the oomycete *Pythium ultimum* in soil, reaching sites in soil that were contaminated with phenanthrene [1]. In another study, it was found that the saprotrophic fungus *Lyophyllum* sp. strain Karsten mediates the migration of *Paraburkholderia terrae* BS001 [2] as well as several other *Paraburkholderia* strains [3] in soil. In this context, we recently affirmed that *P. terrae* BS001 critically relies on flagellum-driven swimming motility for its “forward” dispersal along with fungal hyphae [4]. In contrast, the presence of a type 3 secretion system (T3SS) or of type 4 pili (T4P) was not critical to bacterial co-migration with the fungus, as these systems merely enhanced the flagellar-driven dispersion along fungal hyphae [4, 5].

Bacterial (flagellar) motility along surfaces is affected by key local conditions. First, the hydration status of the surface may be the most important driver. Thus, wet environments, in which sufficiently thick water films occur on local surfaces, have been found to facilitate bacterial movement [6, 7]. Second, the pH at surface microsites may be crucial. As flagellar motility has been reported to be driven by either the proton-motive or the sodium-motive force [8, 9], bacterial translocation may be spurred (on semi-solid agar) by pH decreases, as shown in recent reports [4, 10]. On another notice, factors that drive chemotaxis (e.g., particular fungal exudates) have also been found to modulate bacterial motility [11, 12], with an abundant local nutrient supply suppressing such movement [13].

Most of the aforementioned studies were conducted under laboratory conditions, using artificial media. Indeed, how soil pH and moisture content influence the degree of bacterial motility along mycelial networks has not been well elucidated yet. In particular, *P. terrae* strain BS001 has been reported to migrate in the canonical fungal growth direction and not in the opposite one [2]. This observation has constituted a basis for our model that describes this migration [4]. The model was based on assumptions of aged mycelium becoming, in some way, hostile to *Paraburkholderia* cells migrating along the fungal highway, due to either a changed cell surface or the lack of released (cell-attracting) compounds. In the current study, we critically examine the migration behavior of *P. terrae* with *Lyophyllum* sp. strain Karsten in the canonical (forward) and counter-canonical (backward) direction, as affected by soil pH and soil moisture content. We hypothesized that soil pH, next to moisture content, critically influences the degree of bacterial flagellar movement along with fungal hyphae through soil. In particular, we reasoned that lower soil pH might spur proton-motive force-based flagellar movement. In contrast, soil pH might also act as factor that limits bacterial survival or fitness. Therefore, we surmised that a critical balance exists between (positive) soil pH effects on proton-motive force-driven motility and those (negative) on bacterial fitness. We thus investigated the potential relationship of soil pH with bacterial cell motility in the mycosphere using a well-established three-compartment petri dish based soil microcosm [2, 4, 5].

## Materials and Methods

### Strains and Cultural Conditions

*Paraburkholderia terrae* BS001 wild-type and mutant strains were used in this study. All strains were cultured at 28 °C in Luria-Bertani (LB) broth (Sigma-Aldrich Co., USA), with shaking, or on R2A agar (Difco, USA). The wild-type strain was originally isolated as a co-migrator from the mycosphere of *Lyophyllum* sp*.* strain Karsten (DSM2979) [2]. Three mutant strains were derived from it. These were *P. terrae* BS001*ΔsctD* (mutant in the T3SS [5]), *P. terrae* BS001*ΔpilN* (mutant in T4P [4]), and *P. terrae* BS001*ΔfliF* (mutant in the flagellar system [4]). The fungal host used in this study was the aforementioned *Lyophyllum* sp*.* strain Karsten DSM2979. It was grown on oat flake agar (OFA, 30 g oat flakes, 15 g agar, 1 L H_2_O [2]) at 28 °C. Once every 4 weeks, the fungal strain was transferred to fresh OFA for maintenance.

### Soil Microcosms and Experimental Setup

For all experiments, soil from an agricultural field in Buinen, the Netherlands, was used (denoted B soil). The B soil was characterized as a loamy sand soil, with pH 5.3 and an organic matter content of 3.8% [3]. For some experimental treatments, the soil was adjusted to reduced pH values (about 4.6, 4.2, and 3.8) by adding different amounts of 0.2 M H_2_SO_4_ and subsequent mixing. Then, soils were autoclaved (121 °C, 27 min) for three times, with intermittent incubation at room temperature. The maintenance of the different pH values was confirmed following the last autoclaving before incubation. Soil pH was measured in water (soil/water = 1:5, *w*/*v*). Following these soil pH modulations, soil microcosms were prepared, using three-compartment petri dishes in accordance with previous studies [2, 4, 5]. Briefly, one of the compartments was filled with OFA, and the other two with either one of the pretreated B soil portions (adjusting soil moisture content to 12 or 17%, corresponding to 42 or 60% of water holding capacity [WHC], measured before the soil microcosm preparation to make the experimental conditions consistent). Using the soil microcosms with differently set pH (5.3; 4.6; 4.2; 3.8) and moisture content (12, 17%) values, OFA plugs containing fungal growth were placed in the OFA compartments, and the systems were incubated at 28 °C. Control systems received no fungus. Following incubation, with fungal growth reaching up to 20 (or 30) mm into the soil compartments, bacterial cells were introduced either at the tip of fungal growth or in the middle of the hyphal growth area. Specifically, in experimental set 1, the bacterial cells were introduced at the tip of fungal growth when this reached 20 mm into the soil. In experimental set 2, the bacterial cells were introduced 10 mm away from the tip of fungal growth when this tip reached 30 mm into the soil. Specifically, the bacterial strains (separate introductions for all strains used) were introduced at 5 × 10^5^ cells per soil compartment, establishing a 45 × 3 mm (length x width) inoculated soil zone, and systems were incubated at 28 °C. At set times (4, 7, and 15 days after bacterial inoculation), small samples were recovered, by punching out, from the introduction, “backward” (just behind the barrier) and “forward” (hyphal migration front) sites. All samples were suspended in water, shaken intensely (1 min, three times, with 30-s intervals), serially diluted, and spread on R2A agar plates. Following incubation of the plates, colonies were enumerated and CFU numbers per gram soil were calculated. For each experimental treatment, three replicate microcosms were used. The non-fungal controls, examined similarly, consistently revealed the absence of CFUs on the plates.

To assess the *P. terrae* BS001 population dynamics in the B soil, fresh washed suspensions of all strains (BS001 wild type, BS001*ΔsctD*, BS001*ΔpilN*, and BS001*ΔfliF*) were introduced separately into the different microcosms in the absence of fungal hyphae, and population densities were monitored over time.

### Measurement of Length of Fungal Hyphae in Soil Microcosms

The density of the hyphal growth in soil was measured according to the method described by Shen et al. [14]. Briefly, soil microcosms were sampled at the backward and forward sections, at 10 mm by 20 mm from the soil section, which was one third of the petri dish (45 mm in radius). The soil samples were homogenized in Calgon solution [14] and aliquots used for analyses. The total length of the fungal hyphae in the resulting suspensions was thus examined by microscopy. These measurements were first performed when the fungal hyphal tip reached 20 mm into the soil (day 0), followed by measurements done 4, 7, and 15 days afterwards.

### Statistical Analysis of the Data

All data obtained were subjected to analysis of variance (ANOVA). Also, unpaired two-tailed *t* tests were performed in case two treatments were compared. Differences of the means were considered to be significant at *P* < 0.05. Also, all data were analyzed by Classification and Regression Tree (CART) analysis.

## Results

In earlier work, we showed that migration of *P. terrae* BS001 with soil-exploring hyphae of *Lyophyllum* sp. strain Karsten in an acid (pH 4.1–4.5) soil, denoted G, was only detectable in the fungal growth direction [2]. Moreover, cells that did not live in the vicinity of the hyphal network in soil were found to progressively lose viability. Here, we critically examine the tenet that bacterial migration, in a pH-dependent manner, primarily takes place in the fungal growth direction. We used another soil (a loamy sand soil taken from an agricultural field denoted B, with native pH of 5.3), in microcosms. To understand the contribution of the flagellar, the T3SS, and the T4P systems to the survival and movement of strain BS001, we included, next to the wild type, the respective mutant strains of these systems (denoted BS001*ΔfliF*, BS001*ΔsctD*, and BS001*ΔpilN* [4, 5]) in all experiments. The inclusion of the mutants was justified based on previous indications that pH affects the expression and/or function of the T3SS [15–17] and T4P/twitching motility [18, 19], whereas the flagellum is essential for strain BS001 migration in the mycosphere [4]. The cells were introduced at the “tip” or “middle” sites of the hyphal growth front in the soil compartment (denoted introduction site, see “Materials and methods” section). Following incubation of the microcosm systems, these were regularly sampled at two sites away from the introduction sites, coined the forward and backward movement sites.

### Population Dynamics of *P. terrae* BS001 in Bulk B Soil and in the Mycosphere

**Fig. 1:**
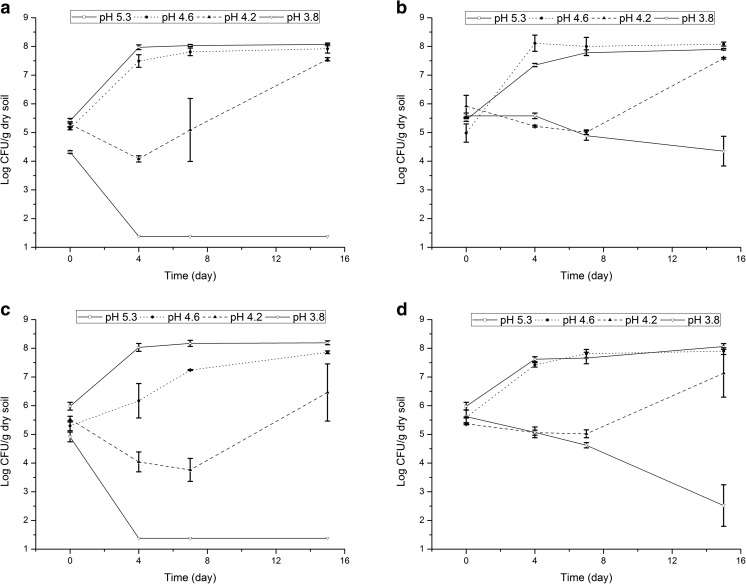
Survival of *P. terrae* BS001 wild type at the introduction site in the *Lyophyllum* sp. strain Karsten mycosphere in B soil. **a** Soil at 60% WHC, tip introduction. **b** Soil at 60% WHC, middle introduction. **c** Soil at 42% WHC, tip introduction. **d** Soil at 42% WHC, middle introduction

Overall, the data showed the *P. terrae* BS001 population sizes to decrease progressively with time, irrespective of strain type (wild type or mutant), in the sterilized B soil in the absence of fungal hyphae. The populations reached the detection limit, of 24 CFU/g dry soil, at day 7 in native (pH 5.3) soil (Fig. S1). Moreover, introduced cell populations lost their viability within 2 days in the soil at pH 4.6 (data not shown) and within 1 day in the soil at the lower pH values (pH 4.2 and 3.8, data not shown). Thus, the B soil, in the absence of fungal hyphae, did not support the survival of strain BS001, and progressive lowering of the pH induced faster decline rates. In contrast, in the presence of fungal hyphae, all bacterial strains survived and—on occasion—even grew at the introduction sites (Fig. 1, Figs. S3–S6).

A full factorial ANOVA performed on all data on the population dynamics at the introduction sites (including the wild type and three mutant strains in the microcosms with soil at all pH and soil moisture levels, Figs. S3–S6) revealed that soil moisture (*F* = 1.073, *P* > 0.1) and strain type (*F* = 0.276, *P* > 0.1) did not significantly influence bacterial survival in the soil (Table S1). In contrast, (initial) soil pH was shown to be a strong determinant of bacterial survival (*F* = 1063.807, *P* < 0.0001), whereas introduction site (tip versus middle of fungal hyphae) was the second important factor (*F* = 79.437, *P* < 0.0001). Time was also a determinant of the abundance of *P. terrae* BS001 CFUs at the introduction sites (*F* = 16.968, *P* < 0.0001). The relative effects of the different factors were also analyzed by CART, as visualized in Fig. S2a and Table S1.

A closer look at the effects of soil pH revealed strain BS001 to survive and even grow in the mycospheres in the pH 5.3 (native) and pH 4.6 soils (Fig. 1). In contrast, its dynamics in the mycosphere in the pH 4.2 soil was erratic, with cell abundances decreasing at days 4 and 7 and increasing at day 15 (Fig. 1). Moreover, the strain BS001 population dynamics in the mycosphere at pH 3.8 clearly depended on introduction site (Fig. 1), with poor survival already after 4 days (tip introduction, Fig. 1a, c) versus survival until day 15 (middle introduction, Fig. 1b, d), even though, in the latter case, cell abundances decreased—with one exception—strongly and significantly (*P* < 0.05) over 15 days (Fig. 1b, d).

### Effects of the Presence of Fungal Hyphae on Soil pH

**Fig. 2:**
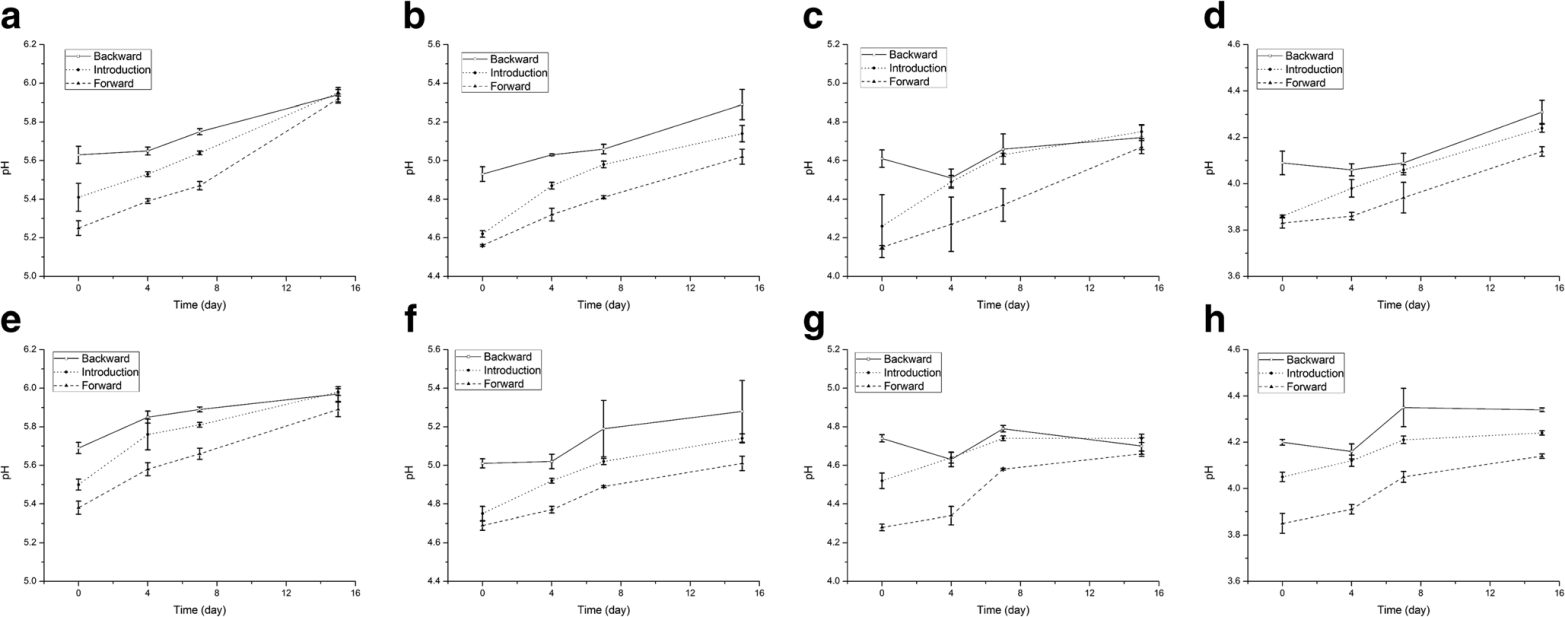
Effect of *Lyophyllum* sp. strain Karsten on local pH in B soil microcosms with bacterial cells (mimicking the condition with bacterial cells introduced at the hyphal growth tip (top row) or at the middle of hyphal growth (bottom row). **a**, **e** Native soil (pH 5.3). **b**, **f** Soil at pH 4.6. **c**, **g** Soil at pH 4.2. **d**, **h** Soil at pH 3.8

In the absence of fungal hyphae, the pH of the soil in the microcosms did not change over the time of the experiment (*P* > 0.05; Fig. S7). In contrast, soil pH was raised—in all cases—in the presence of fungal hyphae, at the introduction and migration sites (*P* < 0.05) (Fig. 2). For instance, the native-pH soil revealed a pH shifting from initially 5.25 ± 0.05 (bulk soil)—5.41 ± 0.09 (experiment 1; introduction site) to 5.95 ± 0.02 at day 15 (*P* < 0.01, Fig. 2a), or from 5.50 ± 0.03 (introduction site, experiment 2) to 5.98 ± 0.02 (*P* < 0.001, Fig. 2e). Similar results of fungal-induced progressively increasing soil pHs were found for the other soils with pHs set at 4.6, 4.2, and 3.8 (Fig. 2b–d, f–h). In these, soil pH increases from 4.56 ± 0.01 to 5.29 ± 0.10, from 4.15 ± 0.01 to 4.75 ± 0.05, and from 3.83 ± 0.03 to 4.34 ± 0.01 were found, respectively.

### Migration of *P. terrae* BS001 in the Mycosphere of *Lyophyllum* sp. Strain Karsten in B Soil

**Fig. 3:**
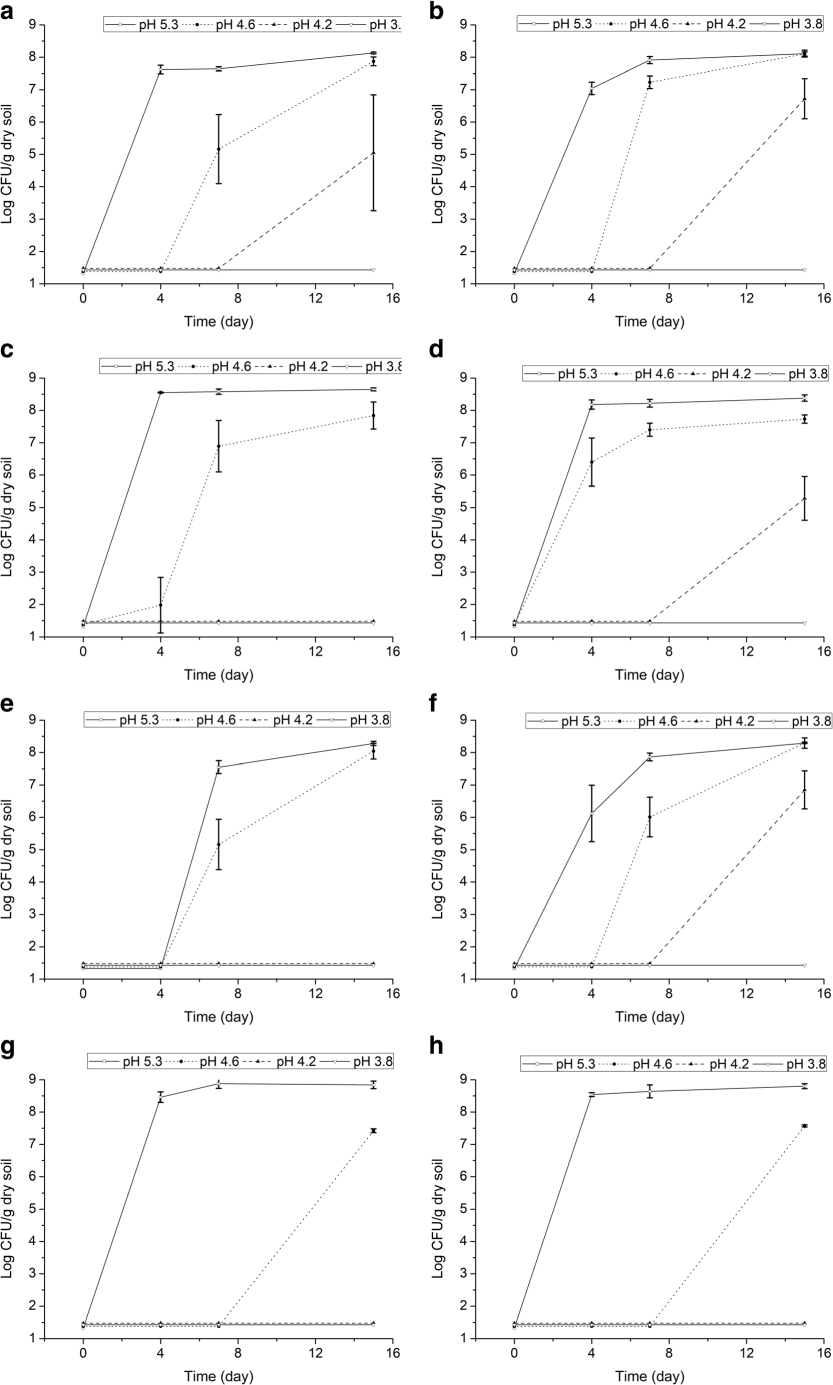
Migration of *P. terrae* BS001 wild type in the *Lyophyllum* sp. strain Karsten mycosphere in B soil. Bacterial cells introduced at hyphal tip (left column) or in middle of hyphal growth (right column). **a**, **b** Backward site, soil at 60% WHC. **c**, **d** Forward site, soil at 60% WHC. **e**, **f** Backward site, soil at 42% WHC. **g**, **h** Forward site, soil at 42% WHC

In the native-pH soil microcosm (pH 5.3), movement of the flagellar-negative mutant along with the growing *Lyophyllum* sp. strain Karsten hyphae was never detected. This confirmed the key relevance of a functional flagellar apparatus for migration [4]. In subsequent work, we thus used this mutant as the negative control of migration. In contrast, all flagellum-positive strains (wild type, BS001*ΔsctD*, and BS001*ΔpilN*) were found to translocate in the canonical growth direction (forward) of the developing hyphae, reaching similar elevated population densities at the hyphal fronts (Fig. 3c). Very surprisingly and contrary to our expectations, consistent migration of these strains in the backward direction (against the direction of fungal growth) was also noticed in these systems (Fig. 3a).

Starting from the premise that soil pH will affect the forward/backward migration of strain BS001 with fungal hyphae (next to affecting bacterial fitness), we then tested the strain BS001 migrational behavior with *Lyophyllum* sp. strain Karsten hyphae in the B soil at different pH values (at both 42 and 60% of WHC). The overall ANOVA of the CFU counts showed, for all three strains (wild type and two mutant strains, in the soil microcosms at all pH and water moisture levels, Figs. S3–S5), an absence of differences between the CFU numbers of the three strains at the forward versus backward migration sites (*F* = 1.632, *P* > 0.05). Thus, the *sctD* or *pilN* mutations did not affect single-strain migrational behavior as compared to that of the wild-type strain (*F* = 0.543, *P* > 0.05). The ANOVA further affirmed that soil pH was the main effector of the bacterial population densities resulting from migration in the mycosphere (*F* = 2179.117, *P* < 0.0001). Other drivers of these densities were time (*F* = 155.202, *P* < 0.0001), soil moisture content (*F* = 24.764, *P* < 0.0001), and introduction site (*F* = 14.589, *P* < 0.001). In contrast, sampling (forward/backward) site was not an important driver, indicating that similar strain BS001 population densities reached the forward and backward sites. Similarly, CART analysis revealed soil pH to drive the first split and time the second (Fig. S2b, Table S1). Figure 3 shows the dynamics of migration of *P. terrae* BS001 wild type in the mycosphere. Extensive data on the migration dynamics of all bacterial strains in the mycosphere can be found in Figs. S3–S5.

The data further revealed that strain BS001 cells reached the migration sites (forward or backward) in the pH 4.6 soil (Fig. 3) later than in the native-pH soil (Fig. 3). Moreover, the population densities at the migration sites were greatly restricted in the pH 4.2 (Fig. 3) and pH 3.8 soils. In the pH 4.2 soil, poor migration was noted (Fig. 3a, b, d, f), whereas populations resulting from migration were not found in the pH 3.8 soil.

Time as a driver of the BS001 population dynamics in the mycosphere was particularly relevant for the pH 4.6 (Fig. 3) and pH 4.2 (Fig. 3a, b, d, f) soils. Clearly, the population sizes were positively related to time under these conditions. Thus, whereas bacteria were detected early on in the native-pH mycosphere (day 4, Fig. 3), they were only detected later in both low-pH mycospheres (day 7 to day 15 in the pH 4.6 and pH 4.2 mycospheres, Fig. 3).

Finally, soil moisture level also affected bacterial migration, in particular cases. In the native-pH soils with soil moisture at 42% of WHC, migration—although ultimately found—was significantly (*P* < 0.05) retarded as compared to the 60% WHC systems (tip introduction; Fig. 3a, e). In the pH 4.6 soils, the forward sites were reached earlier in soil with moisture level at 60% of WHC than in that with moisture at 42% WHC [day 4 (Fig. 3c, d) versus day 7 (Fig. 3g) or day 15 (Fig. 3h)].

### Dynamics of Development of Hyphal Density in Soil (Forward and Backward Directions)

**Fig. 4:**
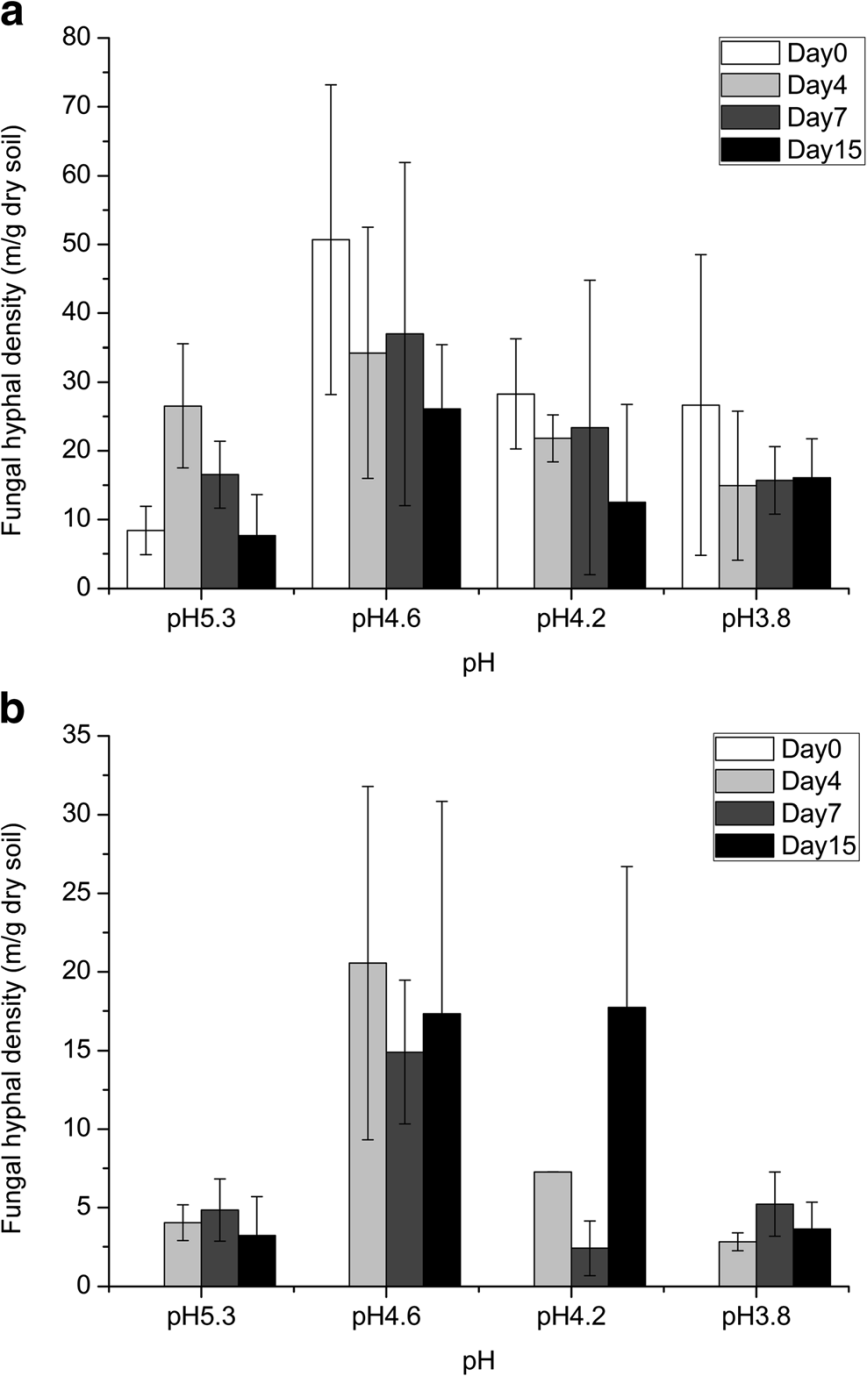
Hyphal density of *Lyophyllum* sp. strain Karsten in B soil. **a** Backward section. **b** Forward section

Importantly, in native-pH soil (pH 5.3), the hyphal density in the backward direction increased from 8.39 ± 3.53 (m/g dry soil) at day 0 to 26.52 ± 9.01 (m/g dry soil) at day 4, after which it progressively decreased (Fig. 4a). This indicated the formation of novel fungal tissue over time in the older hyphal parts. However, in soils with progressively lower pH (4.6, 4.2, and 3.8), the hyphal densities were already high at the early phases (day 0) of the experiment (50.69 ± 22.49 m/g dry soil at pH 4.6, 28.27 ± 7.99 m/g dry soil at pH 4.2, and 26.65 ± 21.87 m/g dry soil at pH 3.8). These densities decreased progressively at the later sampling times (*P* > 0.05, Fig. 4a). In contrast, at the forward site, hyphal densities increased from day 0 to day 7 in native pH as well as pH 3.8 soil (Fig. 4b). In the pH 4.6 and pH 4.2 soils, the respective hyphal densities increased until day 4, after which they fluctuated slightly (Fig. 4b).

### Hyphal Density in Soil, pH Alteration, and Bacterial Movement

As described above, the presence of living *Lyophyllum* sp. strain Karsten hyphae was essential for soil pH alteration. Moreover, it spurred bacterial survival as well as migration in soil. An overall analysis could not detect any positive or negative relationships between the soil hyphal density values and soil pH changes (pH at specific time points versus initial pH values, Fig. S8). Remarkably, when the backward site bacterial CFU data (at pH values 4.6 and 4.2) were analyzed against the (previous) fungal biomass measurements (backward), a weak correlation was found (*R*^2^ = 0.21, Fig. S9), providing evidence for the contention that an increase in the density of hyphae was consistent with the enhanced bacterial motility in these low-pH soils.

## Discussion

Migration of bacteria along with fungal hyphae has received increased focus in recent years, using artificial (non-soil) conditions or soil microcosms. On agar surfaces, *Pseudomonas putida* KT2440 was found to disperse along with the hyphal network of *Morchella crassipes* via flagellar-driven motility [20]. Migration of *Achromobacter* sp. SK1 along with hydrophilic *Fusarium oxysporum* Fo47, allowing to cross air gaps, was also reported [21]. In soil, translocation of *Pseudomonas putida* PpG7 along with hyphae of the oomycete *Pythium ultimum* was reported [1]. In our laboratory, migration of *Paraburkholderia* spp. along with growing fungal hyphae in soil has been reported in several papers [2, 3, 22]. In other recent work, it was found that the soil fungus *Trichoderma* sp. even mediated the movement of eight bacterial types over an air gap between two agar surfaces [23]. However, although we now understand that particular bacteria in soil make use of the networks provided by soil fungi, how soil pH and moisture affect the translocation of such bacteria along these hyphae has not been elucidated yet. Thus, a thorough study on the effects of the soil fungus *Lyophyllum* sp. strain Karsten on the survival and migration of *P. terrae* BS001 in the agricultural B soil was performed. Here, we focused exclusively on the effects of *Lyophyllum* sp. strain Karsten on strain BS001 and derivatives, thus including no-fungus controls. Hence, fungus-only controls were deemed less relevant for the purpose of this study. The data indicated key effects of the initial soil pH on bacterial dynamics in both fungal and non-fungal systems. Clearly, the bacterial population size measurements made by us, as CFUs per gram soil, per site, were the net result of bacterial migration, survival, growth, and death, and hence, we determined the “end” effects of these diverse factors. Both ANOVA and CART analyses (CART, a method from the medical field that develops decision trees for diagnostic classification) [24] showed that that initial soil pH was the main factor that affects strain BS001 survival and migration in the B soil mycosphere.

### Bacterial Survival Is Impaired in B Bulk Soil and Fostered by the Presence of Fungal Hyphae

In the current study, all BS001 strains tested revealed impaired survival in the pre-sterilized B soil, at all pH values, in the absence of fungal mycelium. This rather surprising observation may indicate that the sterilization process (threefold autoclaving) incited conditions in the soil that are, in an as-yet-unknown manner, restrictive to the incoming strain BS001 populations. Irrespective of the nutrients released by the sterilization process, bacterial survival in soil is determined by, next to soil pH, factors such as soil textural type, soil nutrient availability, soil moisture, soil pore size distribution, and the presence of toxic chemicals [25, 26]. We posit that the most plausible explanation for the poor survival may have lied in the release of (unknown) toxic compounds. However, it is possible that bacterial cells become “dormant” under the soil conditions, thus escaping detection by plating; under such conditions, they may have retained their viability. To allow maximal time for outgrowth, in this study, the inoculated R2A agar plates were kept for up to 2 weeks to examine colony growth, albeit to very little avail. In contrast, the presence of *Lyophyllum* sp. strain Karsten clearly protected strain BS001 from the potentially hostile conditions in the B soil, as bacterial survival was strongly promoted, in a pH-dependent manner. The protective effect was akin to that previously found for the G soil. Although we lack evidence for this, it is possible that the fungus—by providing nutrients such as glycerol and oxalate [12, 27]—furnished energy and carbon sources to the inoculant cells which subsequently were able to better establish and survive in the system. The effect regarding the pH raises is discussed below.

### Strain BS001 Migrates Along Hyphae of *Lyophyllum* sp. Strain Karsten in the Backward Direction

In all previous studies in our laboratory, migration of strain BS001 against the growth direction of *Lyophyllum* sp. strain Karsten (backward) could never be detected in the low-pH Gieterveen soil (G soil, pH 4.1–4.5) [2, 3, 22]. Hence, we surmised that the presence of young, actively growing fungal mycelium is a prerequisite for strain BS001 movement along with the hyphae. A mechanistic model, invoking several types of interactive events taking place at the fungal growth tip was developed on the basis of this essential observation [4, 28]. A review of key studies on the migration of other bacteria along with fungal hyphae also did not clearly provide information on the direction of migration [28]. Here, to our surprise, we found that all flagellated forms of *P. terrae* BS001 were able to migrate (in addition to the fungal growth direction) in the non-growth direction of *Lyophyllum* sp. strain Karsten in the B soil, whereas the non-flagellated mutant was not. This migration occurred at (initial) soil pH values as low as 4.2. Previous data from studies on semi-solid agar media have shown that flagellar motility is isotropic [4], but tropic in the presence of fungal hyphae [29]. On the basis of the observation of backward migration, we hypothesized that migration in the mycosphere in the B soil was, in this case, either uncoupled from the fungal tip tropism [2], or that development of new mycelia in the B soil had occurred in the backward regions containing “old” mycelium. Supportive of this latter tenet is the finding that the fungal density indeed increased at the backward site from day 0 to day 4 in the pH 5.3 soil (Fig. 4a), allowing a degree of tip tropism in that region. Moreover, hyphal density development in the backward regions (pH 4.6 and pH 4.2 soils) was found to be correlated (albeit weakly) to the backward migration by strain BS001 found. Consistent with findings by Haq et al. [29], the migration by strain BS001 at *Lyophyllum* sp. strain Karsten might be mediated by chemotaxis towards glycerol or oxalate. Alternatively, the movement in the backward direction might have had another directional driver, possibly including old/senescent hyphae leaking nutrients. In previous studies, movement at old fungal hyphae has been reported for *Achromobacter* sp. SK1 in its interaction with *Fusarium oxysporum* Fo47 [21]. In an ecological sense, the capability to move in both directions along fungal hyphae extends the suite of colonizable niches that are reachable by bacteria that take profit of a fungal network in soil for their migration and exploration of soil habitable sites. This metapopulation-promoting effect potentially enhances overall bacterial population fitness.

### The Size of the Strain BS001 Populations Translocated Along Fungal Hyphae Is Inversely Related to Soil pH

Remarkably, the relationship between the population densities of strain BS001 at the migration sites and the soil pH was inverse to our expectations. A first observation was that, for all initial soil pH values, the pH in the mycosphere increased along with fungal growth in the B soil. This was consistent with findings from an earlier study in G soil [30], and suggested that the hyphae of *Lyophyllum* sp. strain Karsten secrete compounds into the surrounding soil that raise the pH [30]. In all cases, upward soil pH shifts were noted, pointing to similar releases at different initial pH values.

A separate experiment performed on semi-solid agar revealed that *P. terrae* BS001 exhibits stronger swimming activity at lower (5.2) than at higher pH levels (6.0, 6.8, and 7.5). This was attributed to the fact that the motor proteins, that drive flagellar movement, are powered by the proton motive force [4]. However, in the presence of fungal hyphae in soil, bacterial cells reached the migration sites (forward and backward) generally later at the lower than at the higher pre-set pH values. Two processes, which work in an opposite fashion in terms of pH dependency, might have driven the observed population densities, i.e., (1) general pH-driven toxicity and (2) the proton-motive force. With respect to general pH toxicity, at pH levels < 5.0, proton concentrations may be progressively more deleterious to strain BS001 cells, thus reducing overall survival. In soil with the lower pH values, strain BS001 cells may therefore have shown enhanced death (and overall reduced growth) rates (or even no growth in the mycosphere in pH 3.8 soil) as compared to soil at higher pH. Thus, “early” events that drive the cell densities at the introduction sites likely strongly influence those found at the corresponding migration sites (Fig. S10). Apparently, pH toxicity affected strain BS001 survival directly and—via this effect—the outcome of migration along with fungal hyphae. Although at low soil pH the proton-motive force may have been high, the resulting pH toxicity may have restricted bacterial cell densities to a too large extent, thus overriding the proton motive force effect.

### Role of Flagella, T3SS, and T4P in the Migration of Strain BS001 in the Mycosphere

In previous work, the T3SS has been shown to be involved, as a cellular appendix, in the attachment of strain BS001 to fungal hyphae [29], whereas the T4P system has been related to either attachment or twitching motility [4]. Recent work in our lab demonstrated that both systems enhance, but are not essential for, the bacterial migration along with fungal hyphae [4, 5]. In contrast, the presence of functional flagella was found to be essential [4], which was here confirmed and extended to migration against the canonical growth direction of the fungal soil colonizer. Given that local pH can regulate the expression/function of the T3SS and T4P [15, 16, 19], we here included the respective mutant strains to examine whether these systems would show any major effect in the soil pH range tested here. Overall, and in concordance with the previous study [4, 5], both mutant strains showed patterns of single-strain migration in the mycosphere that were akin to those of wild-type strain BS001, indicating that the T3SS and T4P do not have major roles in the migrational/survival responses in the mycosphere. Overall, the roles of the T3SS, T4P, and flagella in strain BS001 migration with fungal hyphae were consistent in the B soil pre-set at various soil pH values.

### Model that Explains the Migration Patterns of Strain BS001 with Fungal Hyphae

To explain the observations discussed in the foregoing, we here propose a model in which the final strain BS001 population densities in soil are strongly determined by the very events that take place directly following introduction. Thus, the cells of strain BS001, upon introduction into the mycosphere, immediately perceive, and respond to, the local conditions. A key immediate response is the allocation of energy (obtained from the local environment in which the fungal counterpart is a major component) to physiological processes that foster local establishment, survival, and possible growth. Motility may be inhibited in this case. The initial events thus may allow the inoculant cell population to establish—to some degree—viable adapted cell populations at the introduction site(s). As time progresses, the cell population may grow, which restricts the overall energy supply per cell, and so, a cue may appear in the population that activates flagellar-mediated translocation of cells along with the fungal hyphae. Mycosphere factors such as released carbon sources like glycerol and oxalate, and tip outgrowth providing anchoring or target sites, may have played key roles. The final outcome of the local processes would be the persistence of a population at maximal cell density at the local introduction site, in addition to the spread of “explorer cells” along with the fungal hyphae to novel microhabitats. This spread of explorer cells includes the movement into the non-canonical (backward) direction, where we presume new “cues” (potentially resulting from novel tips formed) emerged. The spread process is then followed by the subsequent establishment—and outgrowth—of a population at the (forward as well as backward) migration sites. Thus, the motility of such explorer BS001 cells in the mycosphere may be seen as a behavioral response to initial crowding in the “local mycosphere” site.

### The Effect of Soil Water Content on the Migration of Strain BS001 with Fungal Hyphae

Water content can also influence bacterial dynamics and motility in soil. For instance, *Bradyrhizobium japonicum* was found to disperse up to 7 mm in soil at high water content (80–100% of WHC) via flagellar-driven motility [31]. However, the motility of this organism and other bacteria has been shown to be restricted in bulk soil at lower soil moisture level [2, 32]. In the current study, and consistent with a previous report [2], strain BS001 did not migrate in soil without fungal hyphae. The two water levels that were used theoretically allow reasonable to good water connectivity across the soil aggregates, and, hence, we surmised that there was no trigger for movement of the inoculant population in any specific direction, such as in the case of “guidance” by a fungal highway. The presence of fungal hyphae can indeed bridge soil particle aggregates, with the presence of water films around them facilitating migration. Possibly, a thicker water film was present around fungal hyphae in the soil with higher water content, allowing enhanced strain BS001 swimming. Thus, soil water content also played an important role in bacterial migration.

## Conclusion

In conclusion, this study confirmed that flagellated cells of *P. terrae* strain BS001 can move along with fungal hyphae growing through a loamy sand (B) soil, and provides key evidence for the contention that it can also migrate against the fungal growth direction. Soil pH was found to exert a strong negative effect on bacterial survival and motility in the mycosphere, whereas soil moisture, in the range 42–60% of WHC, had weaker, yet significant, effects. The presence of fungal hyphae provided protection to the introduced BS001 populations for survival in the B soil, potentially by alleviating toxicity or pH stress, and a “two-lane highway” for bacterial cells to migrate. Bacterial migration in the backward direction was weakly related to recently produced fungal mycelium, but not to pH alteration in the soil.

## Electronic supplementary material

ESM 1 (PDF 1231 kb)
