# Supplementary material for: Migration of Paraburkholderia terrae BS001 Along Old Fungal Hyphae in Soil at Various pH Levels
Source: Microb Ecol. 2018 Jan 10;76(2):443–52. doi: 10.1007/s00248-017-1137-1 (PMC6061471; doi:10.1007/s00248-017-1137-1)
Supplement: Supplementary file 1 — (PDF 1231 kb) [file 248_2017_1137_MOESM1_ESM.pdf]

Supplementary Information

Migration of *Paraburkholderia terrae* BS001 along old fungal hyphae in soil at various pH levels

Microbial Ecology

Pu Yang, Renata Oliveira da Rocha Calixto, Jan Dirk van Elsas

Microbial Ecology, Groningen Institute for Evolutionary Life Sciences, University of Groningen, Nijenborgh 7, 9747 AG Groningen, The Netherlands

Corresponding author: Jan Dirk van Elsas, E-mail address: j.d.van.elsas@rug.nl.

Table S1 Factor importance ranking computed by ANOVA and CART

Factor	Survival [a]				Migration [b]			
	Number of factor levels	F value	P value	CART score	Number of factor levels	F value	P value	CART score
pH	4	1063.807	<0.0001	79	4	2179.117	<0.0001	87
Strain type	4	0.276	0.843	-	4	0.543	0.581	
Introduction site	2	79.437	<0.0001	14	2	14.589	<0.01	-
Sampling time	3	16.968	<0.0001	8	3	155.202	<0.0001	13
Sampling site	1	-	-	-	2	1.632	0.202	-
Soil moisture	2	1.073	0.301	-	2	24.764	<0.001	-

a, data from introduction sites

b, data from both forward and backward migration sites

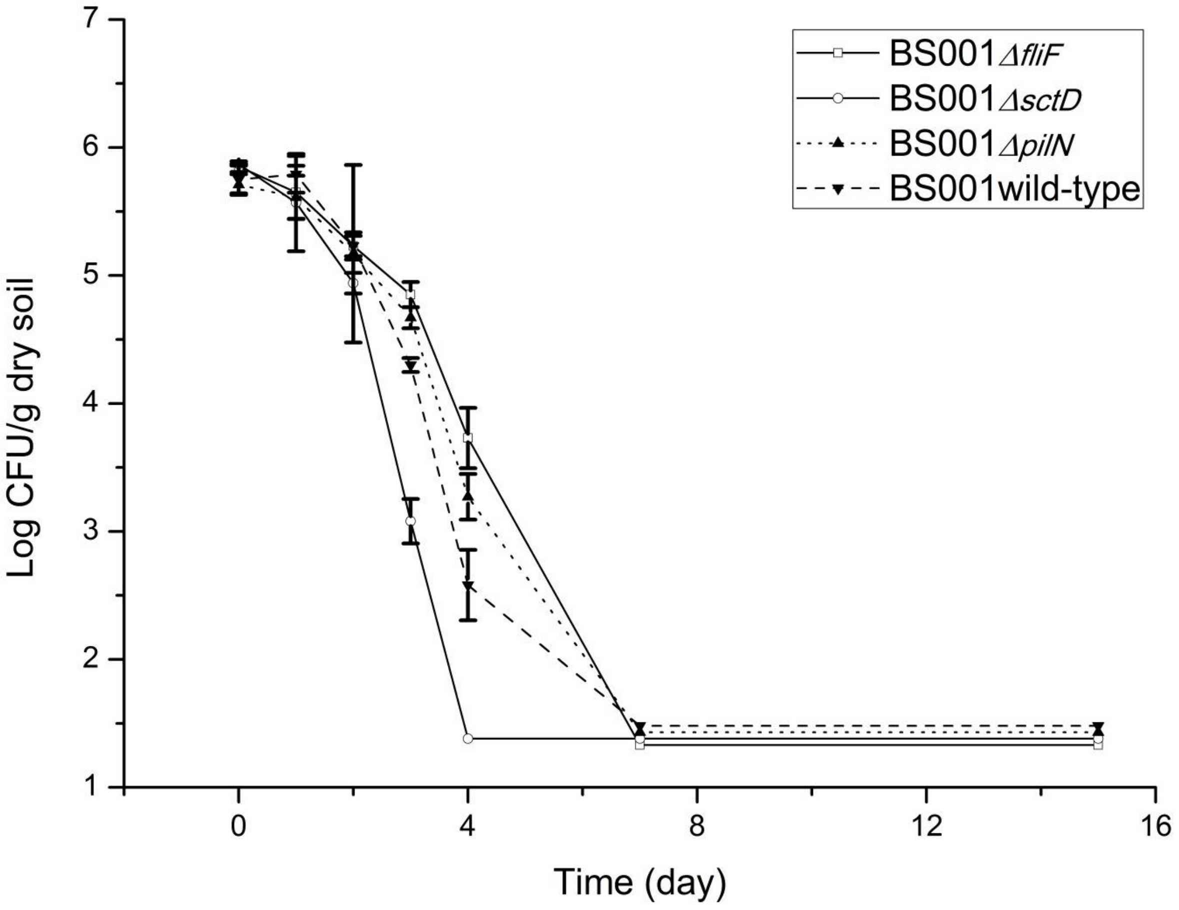

Fig. S1 Bacterial cell survival in native (pH 5.3) B bulk soil without fungal hyphae.

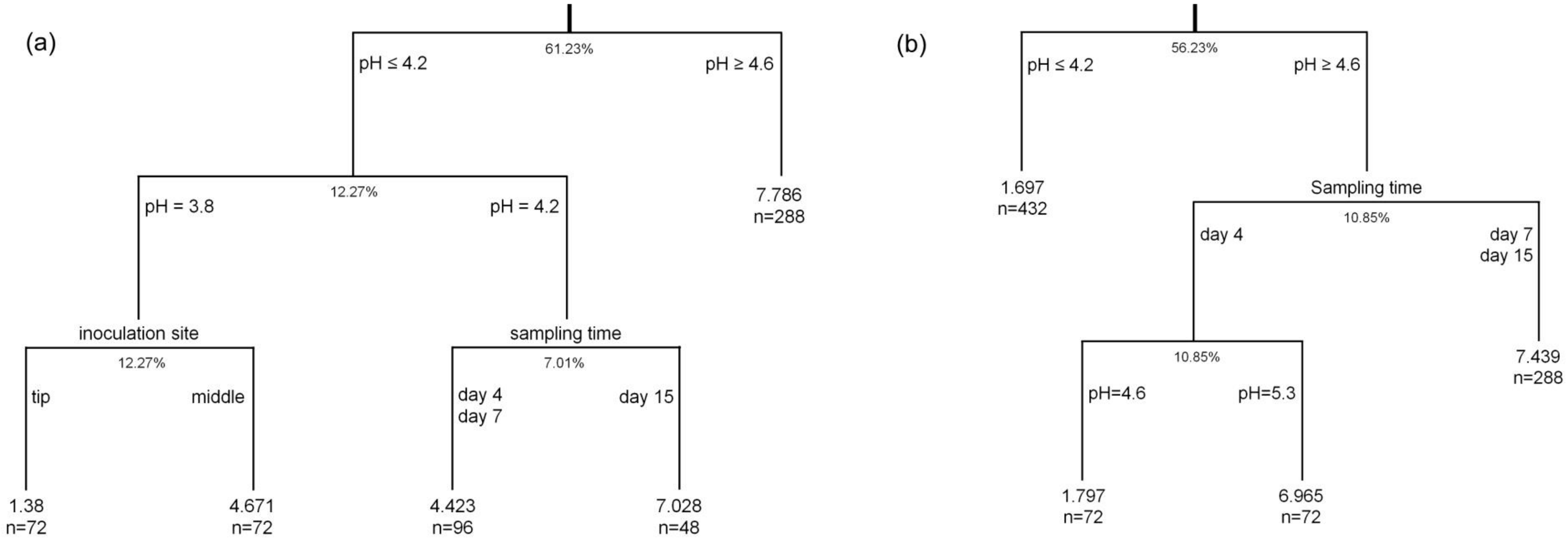

Fig. S2 CART analysis of the factors influencing strain BS001 cell survival (a, data at inoculation site), and migration (b, data from both migration backward and forward sites). The tree at (a) explains 92.62% of the variance in cell survival. Of this, 61.23% was accounted for by the first split, and 12.27% by the second split. The tree at (b) explains 77.38% of the variance in cell migration. Of this, 56.23% is explained by the first split, and 10.85% by the second split.

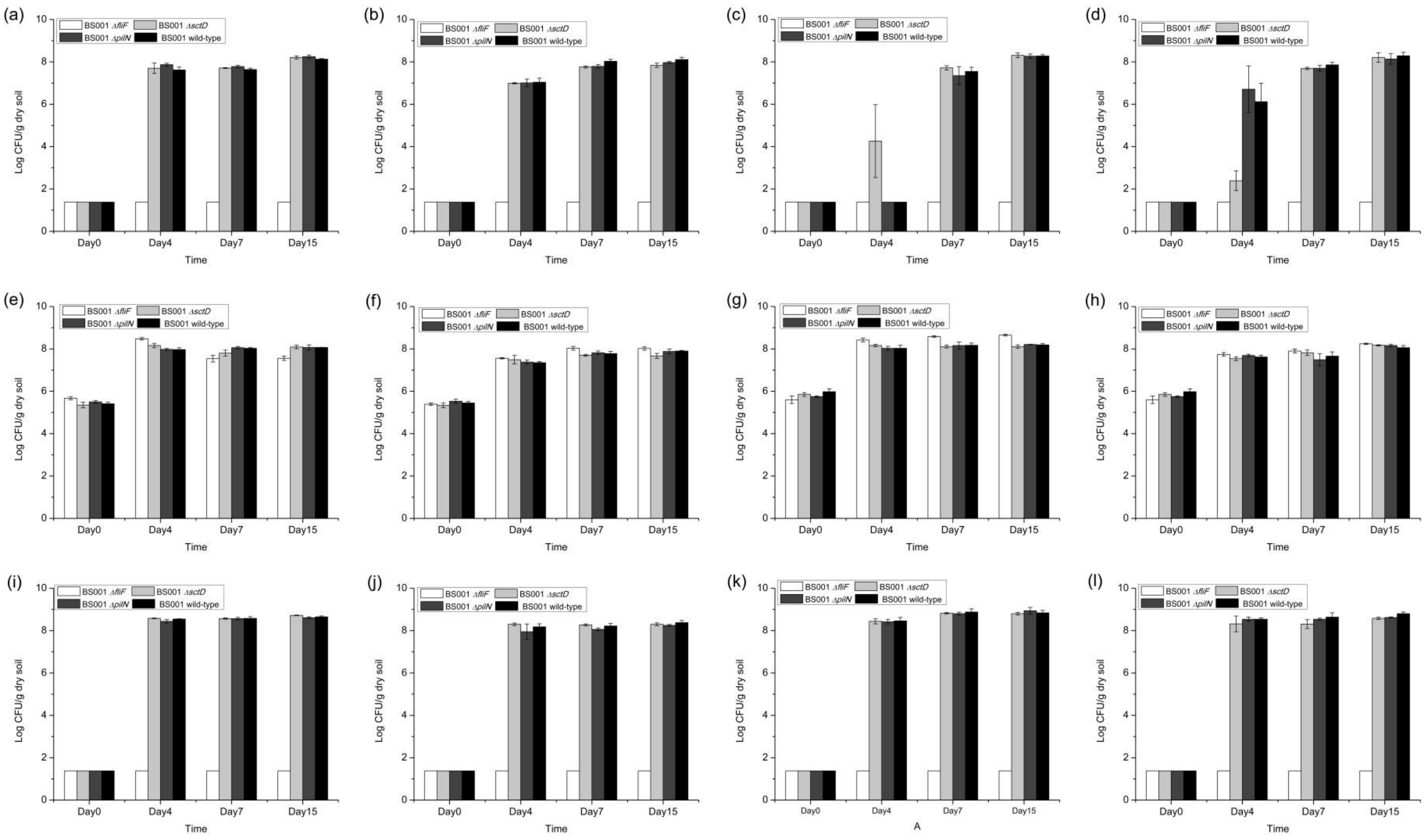

Fig. S3 Bacterial cell (wild-type and 3 mutant strains) abundance in the microcosm with native soil (pH 5.3). Left two columns (a, b, e, f, i, j): 17% soil moisture; right two columns (c, d, g, h, k, l): 12% soil moisture; 1st and 3rd columns(a, e, i, c, g, k): introduced at the tip of fungal growth; 2nd and 4th columns: introduced at the middle of fungal growth; top row (a, b, c, d): backward migration site; middle row (e, f, g, h): introduction site; bottom row (i, j, k, l): forward migration site.

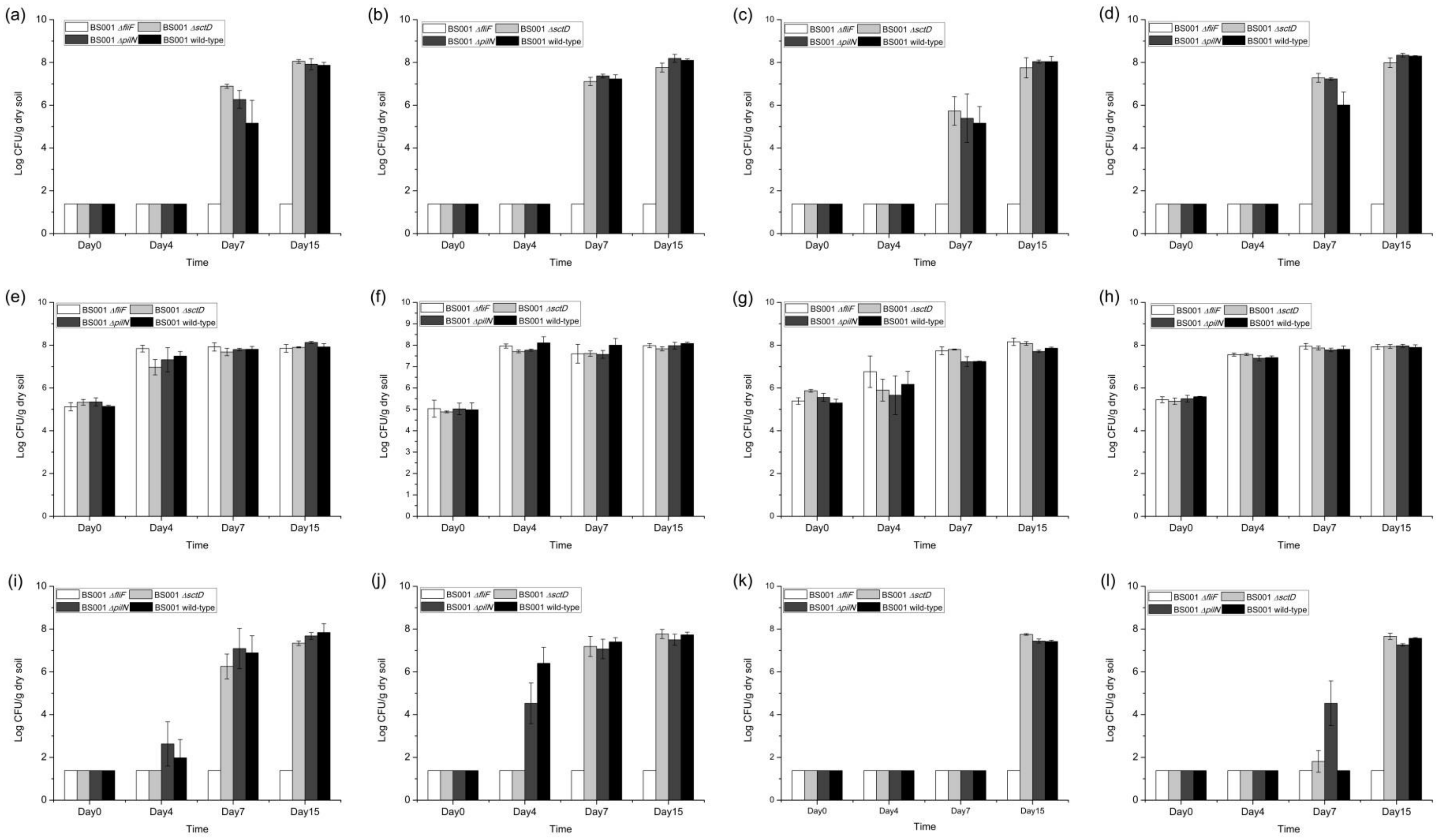

Fig. S4 Bacterial cell (wild-type and 3 mutant strains) abundance in the microcosm with soil at pH 4.6. Left two columns (a, b, e, f, i, j): 17% soil moisture; right two columns (c, d, g, h, k, l): 12% soil moisture; 1st and 3rd columns(a, e, i, c, g, k): introduced at the tip of fungal growth; 2nd and 4th columns: introduced at the middle of fungal growth; top row (a, b, c, d): backward migration site; middle row (e, f, g, h): introduction site; bottom row (i, j, k, l): forward migration site.

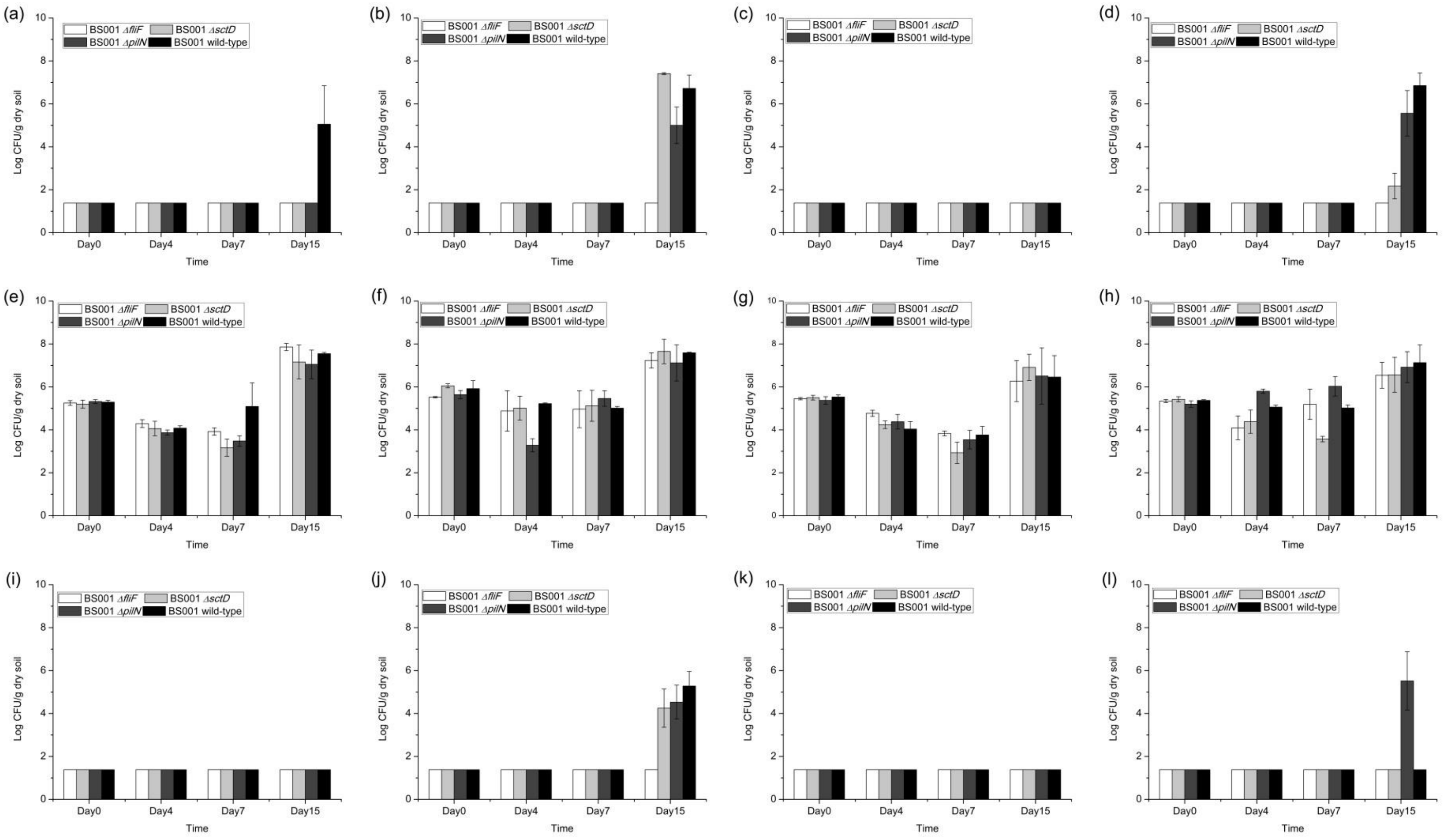

Fig. S5 Bacterial cell (wild-type and 3 mutant strains) abundance in the microcosm with soil at pH 4.2. Left two columns (a, b, e, f, i, j): 17% soil moisture; right two columns (c, d, g, h, k, l): 12% soil moisture; 1st and 3rd columns(a, e, i, c, g, k): introduced at the tip of fungal growth; 2nd and 4th columns: introduced at the middle of fungal growth; top row (a, b, c, d): backward migration site; middle row (e, f, g, h): introduction site; bottom row (i, j, k, l): forward migration site.

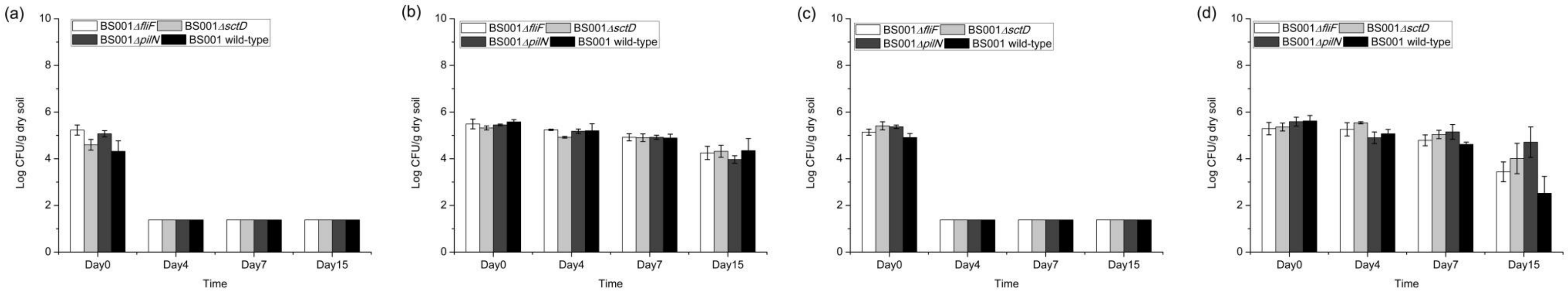

Fig. S6 Bacterial cell (wild-type and 3 mutant strains) abundance at introduction site in the microcosm with soil at pH 3.8. a and b, 17% soil moisture; c and d, 12% soil moisture; a and c, introduced at fungal growth tip; b and d, introduced at the middle of fungal growth.

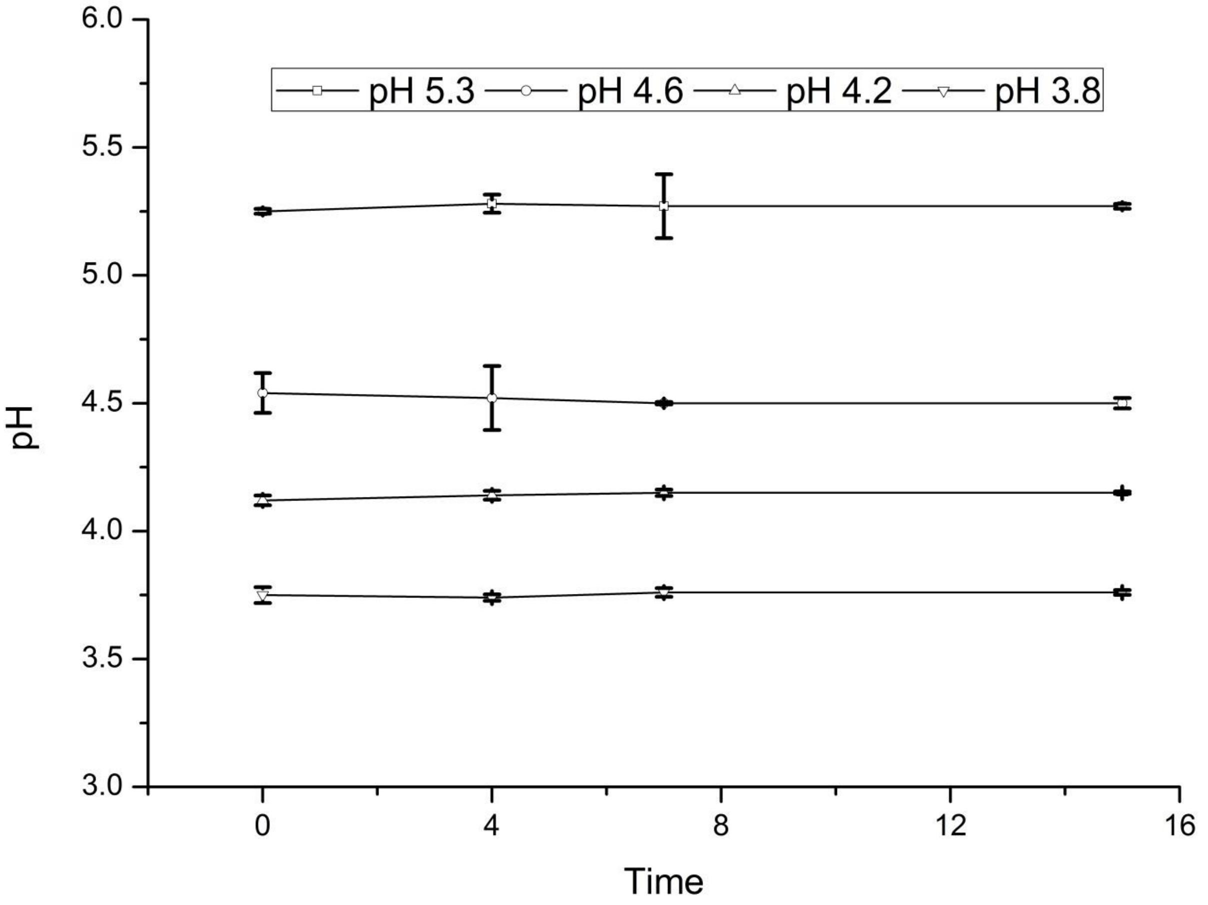

Fig. S7 pH changes in B bulk soil over time (measured in fungus-free soil microcosms)

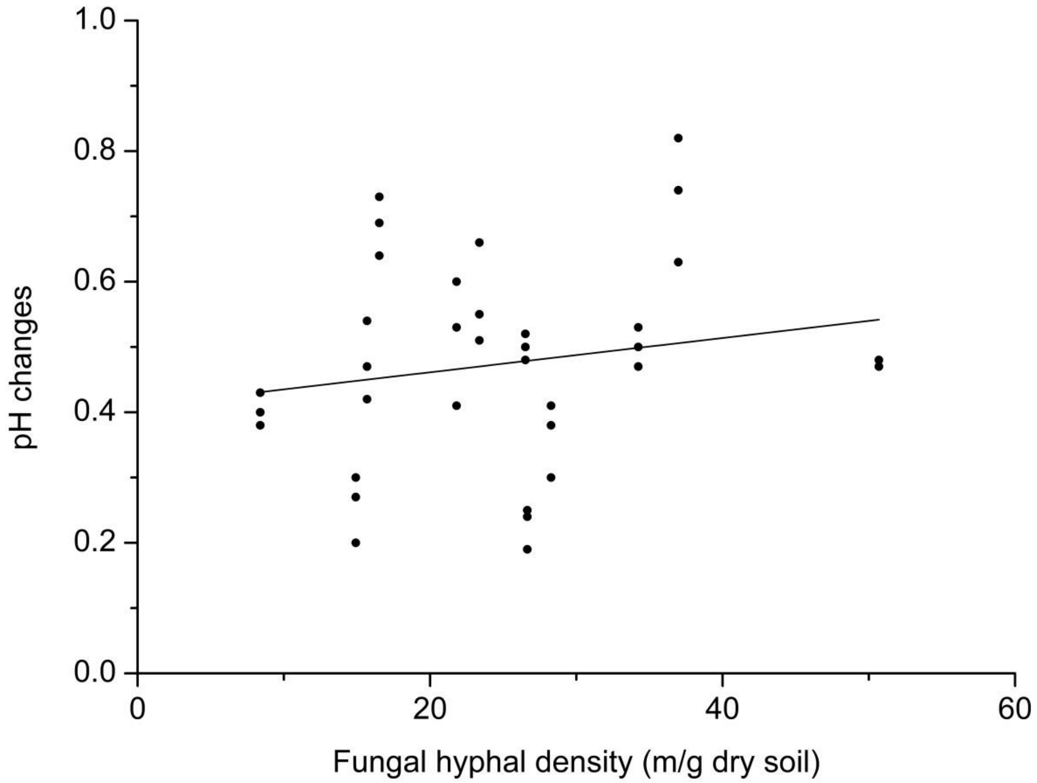

Fig. S8 Correlation between fungal hyphal density and soil pH changes (R^2=0.008)

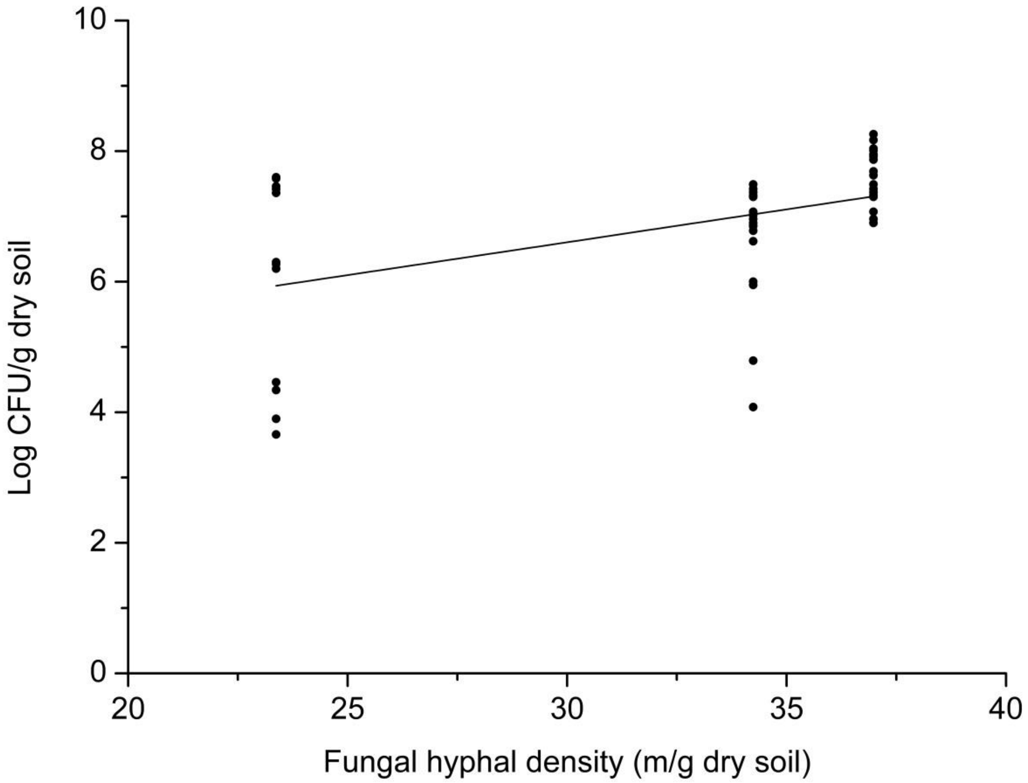

Fig. S9 Correlation between fungal hyphal density in “backward” regions in soil microcosms (pH 4.6 and 4.2 soils) and bacterial abundance (R^2=0.214)

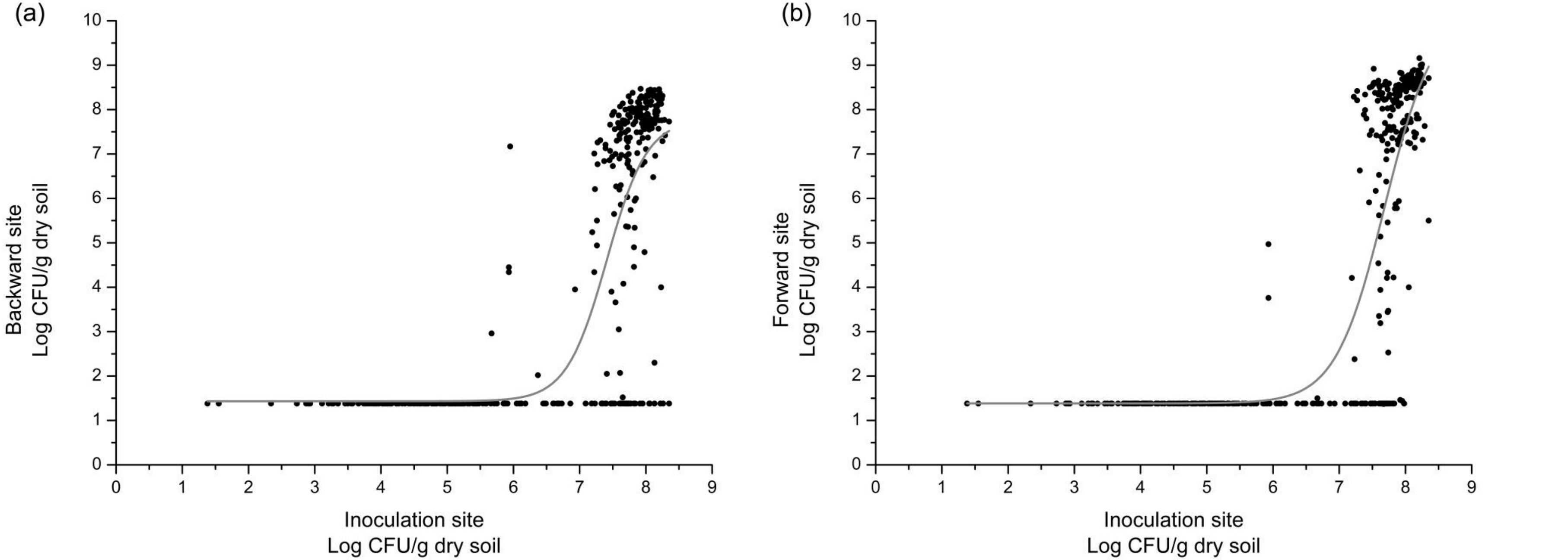

Fig. S10 (a) Relationship of the motile strain (BS001 wild-type strain, BS001*ΔsctD*, BS001*ΔpilN*) CFU counts at inoculation site and backward site (R^2=0.678). (b) Relationship of the motile strain (BS001 wild-type strain, BS001*ΔsctD*, BS001*ΔpilN*) CFU counts at inoculation site and forward site (R^2=0.717).
